# Making sense of “alternative”, “complementary”, “unconventional” and “integrative” medicine: exploring the terms and meanings through a textual analysis

**DOI:** 10.1186/s12906-016-1111-3

**Published:** 2016-05-20

**Authors:** Jeremy Y. Ng, Heather S. Boon, Alison K. Thompson, Cynthia R. Whitehead

**Affiliations:** Leslie Dan Faculty of Pharmacy, University of Toronto, 144 College Street, Toronto, Ontario M5S 3M2 Canada; Women’s College Hospital and Department of Family and Community Medicine, Faculty of Medicine, University of Toronto, and Women’s College Hospital, Toronto, Ontario M5S 1B2 Canada

## Abstract

**Background:**

Medical pluralism has flourished throughout the Western world in spite of efforts to legitimize Western biomedical healthcare as “conventional medicine” and thereby relegate all non-physician-related forms of healthcare to an “other” category. These “other” practitioners have been referred to as “unconventional”, “alternative” and “complementary”, among other terms throughout the past half century.

**Methods:**

This study investigates the discourses surrounding the changes in the terms, and their meanings, used to describe unconventional medicine in North America. Terms identified by the literature as synonymous to unconventional medicine were searched using the Scopus database. A textual analysis following the method described by Kripendorff 2013 was subsequently performed on the five most highly-cited unconventional medicine-related peer-reviewed literature published between 1970 and 2013.

**Results:**

Five commonly-used, unconventional medicine-related terms were identified. Authors using “complementary and alternative”, “complementary”, “alternative”, or “unconventional” tended to define them by what they are *not* (e.g., therapies not taught/used in conventional medicine, therapy demands not met by conventional medicine, and therapies that lack research on safety, efficacy and effectiveness). Authors defined “integrated/integrative” medicine by what it *is* (e.g., a new model of healthcare, the combining of both conventional and unconventional therapies, accounting for the whole person, and preventative maintenance of health). Authors who defined terms by “what is not” stressed that the purpose of conducting research in this area was solely to create knowledge. Comparatively, authors who defined terms by “what is” sought to advocate for the evidence-based combination of unconventional and conventional medicines. Both author groups used scientific rhetoric to define unconventional medical practices.

**Conclusions:**

This emergence of two groups of authors who used two different sets of terms to refer to the concept of “unconventional medicine” may explain why some journals, practitioner associations and research/practice centres may choose to use both “what is not” and “what is” terms in their discourse to attract interest from both groups. Since each of the two groups of terms (and authors who use them) has different meanings and goals, the evolution of this discourse will continue to be an interesting phenomenon to explore in the future.

## Background

Medical pluralism has flourished despite efforts to legitimize Western biomedical health care as “conventional medicine”, thereby relegating non-physician forms of healing to an “other” category. These “other” practitioners have been labelled as “unorthodox”, “unconventional”, “alternative” and “complementary”, among other terms throughout history [1, 2]. The changes in these terms and their meanings are the subject of this inquiry. By exploring the peer-reviewed medical literature discourses with a focus on the individuals and groups that were instrumental in these changes, this study sheds light on how conventional practitioners and researchers understand, label and categorize “others” providing health care to patients.

### “Conventional” medicine

Numerous terms have emerged to identify the type of medicine practised by physicians including: “orthodox”, “allopathic”, “modern”, “scientific”, “bio-”, “evidence-based”, “Western”, “mainstream”, and “conventional”, each preceding the word “medicine”, and sometimes “therapy” [3, 4]. For the purpose of this study, we thought it important that a neutral term, with minimal negative or positive connotation was employed, considering that this study investigates the terminology itself. The word “conventional” was chosen to frame this study because it is both widely understood, common in the literature, and relatively neutral in referring to the politically dominant system of medicine at any historical time point [2]. For the purpose of this study, “conventional medicine” is defined as “medical interventions that are taught extensively at US [and Canadian] medical schools and generally provided at US [and Canadian] hospitals” ([5], p. 246).

### “Unconventional” medicine

We can see that there is great difficulty in defining terms which refer to medical therapies outside the realm of conventional medicine [6]. Over the past half-century, there has not been a single, agreed upon overarching name given to unconventional systems and their practitioners. A variety of terms have been used to describe therapies excluded from conventional medicine including: “irregular”, “unorthodox”, “unscientific”, “quack”, “fringe”, “folk”, “alternative”, “adjunctive”, “alternative and complementary”, “complementary and alternative”, “complementary”, “integrated”, “integrative”, “non-mainstream”, and “unconventional”, each preceding the word “medicine” or “therapy” [1, 2]. Many of these terms, with the possible exception of the terms “complementary” “integrated”, “integrative”, “non-mainstream”, and “unconventional”, suggest a negative connotation associated with the safety and/or effectiveness of these medicines [1]. The words “complementary”, “integrated”, and “integrative”, suggest a certain relationship between conventional medicine and “other” therapies, and thus these terms do not appear to refer to all therapies not included in conventional medicine [7–10].

Similar to the word “conventional”, the word “unconventional” was chosen to frame this study because it is widely understood, common in the literature, and suggests a sense of neutrality when referring to the politically subordinated system of medicine at any point in time [2]. For the purpose of this study, “unconventional medicine” is defined as “medical interventions that are **not** taught extensively at US [and Canadian] medical schools and generally **not** provided at US [and Canadian] hospitals” ([5], p. 246).

Regardless of the terms used to describe unconventional medicine, the challenge of definition still exists [5, 11, 12]. This is partly due to multiple factors that contribute to the reality that a large number of very different unconventional systems and practitioners exist, and thus, any given term must encompass a wide range of practices and beliefs [5]. Unconventional medicines arise from multiple histories, schools of thought, and world regions. Furthermore, some previously unconventional therapies have since been integrated into medical school curricula, at which point they are technically excluded from the categorization as unconventional medicine [12]. Selecting a term with a meaning that accurately accounts for all forms of unconventional therapies and interventions is both complex and dynamic.

### Theoretical framework: scientific rhetoric boundary work

Theories about how groups of health care practitioners form, evolve and interact provide necessary context when aiming to understand the discourses surrounding unconventional medicine. One such theory relevant to this study is that of Thomas Gieryn [13], who asserts that science is often used by those seeking to distinguish and demarcate their work and its products from “non-scientific” intellectual enterprises. This “boundary work” has occurred throughout history, with a notable example being how the miasmatists of the 1800s sought to discredit the work of John Snow, who was sceptical of the miasmatic theory of disease. The famous London cholera outbreak of 1854 eventually lead to the acceptance of a pre-germ-theory-notion of transmission via water, but only after many lives were unnecessarily lost because of the refusal of the miasmatists to acknowledge that John Snow’s data maps were compelling forms of evidence against their theory of cholera transmission through noxious air [14]. Had John Snow not been a well-respected anaesthesiologist, whose patients included Queen Victoria, his theory of cholera transmission would likely never have been given a second look because of his unorthodox epidemiologic methods and wild departure from the dominant scientific understanding of disease that governed public health at the time. Even today, hierarchies of evidence are taught in medical schools, and randomised controlled trials are held out as the most compelling forms of evidence, despite the fact they have limited applicability to real-world settings where patients are heterogenous [15]. Gieryn’s boundary work relating to scientists argues that they construct a social boundary in order to distinguish their intellectual activities from those which are not “scientific” [13]. Gieryn asserts that science serves as a type of intellectual authority because scientific knowledge has come to be widely accepted by society as the preferred truth in describing and explaining reality. Finally, he argues that science is more dynamic and ambiguous than scientists may claim, for it is not a single entity, nor are boundaries constructed similarly, but instead are created in response to challenges faced by different obstacles lying in the way of achieving authority and material resources [13].

Applying Gieryn’s perspective served to focus the inquiry on exploring if, and how, conventional practitioners utilize the rhetoric of science to legitimize practices of their own or delegitimize practices of others. We show that conventional practitioners’ use a scientific notion of “effectiveness” to establish the validity of their therapies and research methods, as a rhetorical strategy to establish their authority. Concomitantly, conventional practitioners label unconventional practitioners and their therapies, “unscientific”, which functions to exclude them from conventional medicine and deny them legitimacy and resources.

### Study aim

The overall purpose of this research study was to conduct a textual analysis to identify the shifts in how unconventional systems (and their practitioners) have been described over time in the peer-reviewed literature applicable to Canada and the United States from 1970 to 2013. The specific objectives of this study were two-fold:

1. to identify changes in the naming of unconventional medicine over time in the peer-reviewed medical literature and;
2. to conduct a textual analysis to explore how changes in the naming of unconventional medicine occurred and who contributed to them.

## Methods

### Study design: textual analysis

Textual analysis is a specific study design derived from content analysis. The latter comprises a group of techniques helpful in analysing and understanding samples of text [16, 17]. Content analysis is one type of study design used in qualitative research, which involves attention to the content of texts and circumstantial meaning, focussing on language characteristics as communication [16, 18–20].

The goal of conducting a textual analysis is to identify hidden meanings, as well as unquestioned patterns and accentuations of texts, where the intent of the researcher is to acquire a deeper understanding of the context in which the text is written. Hence, textual analysis views a text as a “cultural artifact”, and identifies why written work is produced, in addition to the intended audience of the text being analyzed. Textual analysis focusses on repeated patterns, placing, striking imagery, style, and tone, as examples of items which enable researchers utilizing this study design to elicit “the structures of meanings and the configurations of feelings on which this public rhetoric is based” [21]. This study adopted a six-stage textual analysis model based on Klaus Krippendorff’s components of content analysis: unitizing, sampling, coding, reducing, inferring, and narrating [22].

Unitizing guides the researcher in deciding what type of data should be collected and analyzed. For this study, this was limited to the peer-reviewed literature as these articles serve to communicate discussions and controversies concerning researchers’ and practitioners’ understanding of unconventional medicine.

The next step, sampling, involved identifying an appropriate sample of peer-reviewed articles [22], retrieved from the Scopus searches. Sampling allows the researchers to limit observations and analysis, in cases where it is impractical to analyze all texts relevant to the study [22]. In this study, it facilitated a focus on the most commonly used terms.

Coding refers to bridging the gap between texts and the researcher’s reading of them. In this stage, coding categories were developed from the data guided by the theories of professions discussed in the Theoretical Framework section above. According to Krippendorff [22], “researchers can avoid simplistic formulations and tap into a wealth of available conceptualizations” by deriving categories from established theories. Coding is an iterative process. Codes are created and revised as additional data are collected.

Reducing the data helps researchers to efficiently represent large volumes of data, ultimately reducing the diversity of text to what matters through summarizations [22]. Following the initial collection and coding of text excerpts, all excerpts were examined to question if they were grouped into the most appropriate categories to explain the data, and to explore if the categories could be grouped into themes. Original categories or themes may have been removed, merged or split during this review. This process was repeated until all categories and themes had been satisfactorily developed and all excerpts were adequately indicative of each category or theme.

Inferring refers to identifying what unobserved phenomena mean, or refer to, supported by evidence found within the text [22]. This involved examining how the texts in each article were organized and presented and what specific “angle” the authors’ were taking. Additionally, the general “mood” of the text was taken into account, where careful attention was paid to concepts either depicted prominently, unimportantly or omitted altogether, allowing the researcher to identify authors’ presuppositions and subtexts [23].

Lastly, narrating makes the researcher’s findings understandable to everyone else. This stage involved describing the practical significance of the results and how the contributions made by our study impacted the existing literature previously published within this subject area [22].

### Data collection

On September 11, 2014, a series of advanced searches of the Scopus database were conducted, encompassing all terms previously identified as being used to represent unconventional medicine synonyms in the literature [1, 2]. These terms are provided in Table 1.

**Table 1:** Screening of Commonly Used Search Terms for Year of Use Pertaining to Unconventional Medicine

Search Term (Preceding “Medicine” or “Therapy”)	Scopus search code	Explanation of coding	Number of articles recovered
Complementary and Alternative	(TITLE(“complementary and alternative medicine” OR “complementary alternative medicine” OR “complementary and alternative therapy” OR “complementary alternative therapy” AND NOT “integrative” AND NOT “integrated”)) AND PUBYEAR > 1969 AND PUBYEAR < 2014 AND (LIMIT-TO (LANGUAGE, “English”)) AND (LIMIT-TO (SRCTYPE, “j”)) AND (EXCLUDE(DOCTYPE, “er”))	Excludes titles containing “integrative” or “integrated”, in order to prevent other combinations from being searched.	2814
Complementary	(TITLE(“complementary medicine” OR “complementary therapy” AND NOT “alternative” AND NOT “integrative” AND NOT “integrated”)) AND PUBYEAR > 1969 AND PUBYEAR < 2014 AND (LIMIT-TO(LANGUAGE, “English”)) AND (LIMIT-TO (SRCTYPE, “j”)) AND (EXCLUDE(DOCTYPE, “er”))	Excludes titles containing “alternative”, “integrative” or “integrated”, in order to prevent other combinations from being searched.	1758
Alternative	(TITLE(“alternative medicine” OR “alternative therapy” AND NOT “complementary” AND NOT “integrative” AND NOT “integrated”)) AND PUBYEAR > 1969 AND PUBYEAR < 2014 AND (LIMIT-TO(LANGUAGE, “English”)) AND (LIMIT-TO(SRCTYPE, “j”)) AND (EXCLUDE(DOCTYPE, “er”))	Excludes titles containing “complementary”, “integrative” or “integrated”, in order to prevent other combinations from being searched.	1708
Integrated/ Integrative	(TITLE(“integrative medicine” OR “integrated medicine” AND NOT “alternative” AND NOT “complementary”)) AND PUBYEAR > 1969 AND PUBYEAR < 2014 AND (LIMIT-TO (LANGUAGE, “English”)) AND (LIMIT-TO (SRCTYPE, “j”)) AND (EXCLUDE(DOCTYPE, “er”))	Excludes titles containing “alternative” or “complementary”, in order to prevent other combinations from being searched. The word “therapy” was removed to increase the relevance of results.	455
Adjunctive^a^	(TITLE(“adjunctive medicine” OR “adjunctive therapy”)) AND PUBYEAR > 1969 AND PUBYEAR < 2014 AND (LIMIT-TO(LANGUAGE, “English”)) AND (LIMIT-TO(SRCTYPE, “j”)) AND (EXCLUDE(DOCTYPE, “er”))	Standard Code^b^	816
Folk^a^	(TITLE(“folk medicine” OR “folk therapy”)) AND PUBYEAR > 1969 AND PUBYEAR < 2014 AND (LIMIT-TO(LANGUAGE, “English”)) AND (LIMIT-TO(SRCTYPE, “j”)) AND (EXCLUDE(DOCTYPE, “er”))	Standard Code^b^	429
Alternative and Complementary	(TITLE(“alternative and complementary medicine” OR “alternative complementary medicine” OR “alternative and complementary therapy” OR “alternative complementary therapy” AND NOT “integrated” AND NOT “integrative”)) AND PUBYEAR > 1969 AND PUBYEAR < 2014 AND (LIMIT-TO(LANGUAGE, “English”)) AND (LIMIT-TO(SRCTYPE, “j”)) AND (EXCLUDE(DOCTYPE, “er”))	Excludes titles containing “integrative” or “integrated”, in order to prevent other combinations from being searched.	116
Unconventional	(TITLE(“unconventional medicine” OR “unconventional therapy”)) AND PUBYEAR > 1969 AND PUBYEAR < 2014 AND (LIMIT-TO (LANGUAGE, “English”)) AND (LIMIT-TO (SRCTYPE, “j”)) AND (EXCLUDE(DOCTYPE, “er”))	Standard Code^b^	103
Complementary and Integrated/ Integrative	(TITLE(“complementary and integrated medicine” OR “complementary integrated medicine” OR “complementary and integrated therapy” OR “complementary integrated therapy” OR “complementary and integrative medicine” OR “complementary integrative medicine” OR “complementary and integrative therapy” OR “complementary integrative therapy” AND NOT “alternative”)) AND PUBYEAR > 1969 AND PUBYEAR < 2014 AND (LIMIT-TO (LANGUAGE, “English”)) AND (LIMIT-TO (SRCTYPE, “j”)) AND (EXCLUDE(DOCTYPE, “er”))	Excludes titles containing “alternative” in order to prevent other combinations from being searched.	27
Unorthodox	(TITLE(“unorthodox medicine” OR “unorthodox therapy”)) AND PUBYEAR > 1969 AND PUBYEAR < 2014 AND (LIMIT-TO (LANGUAGE, “English”)) AND (LIMIT-TO (SRCTYPE, “j”)) AND (EXCLUDE(DOCTYPE, “er”))	Standard Code^b^	14
Fringe	(TITLE(“fringe medicine” OR “fringe therapy”)) AND PUBYEAR > 1969 AND PUBYEAR < 2014 AND (LIMIT-TO(LANGUAGE, “English”)) AND (LIMIT-TO(SRCTYPE, “j”)) AND (EXCLUDE(DOCTYPE, “er”))	Standard Code^b^	12
Integrated/ Integrative and Complementary	(TITLE(“integrated and complementary medicine” OR “integrated complementary medicine” OR “integrated and complementary therapy” OR “integrated complementary therapy” OR “integrative and complementary medicine” OR “integrative complementary medicine” OR “integrative complementary therapy” OR “integrative complementary therapy” AND NOT “alternative”)) AND PUBYEAR > 1969 AND PUBYEAR < 2014 AND (LIMIT-TO (LANGUAGE, “English”)) AND (LIMIT-TO (SRCTYPE, “j”)) AND (EXCLUDE(DOCTYPE, “er”))	Excludes titles containing “alternative” in order to prevent other combinations from being searched.	8
Quack	(TITLE(“quack medicine” OR “quack therapy”)) AND PUBYEAR > 1969 AND PUBYEAR < 2014 AND (LIMIT-TO(LANGUAGE, “English”)) AND (LIMIT-TO(SRCTYPE, “j”)) AND (EXCLUDE(DOCTYPE, “er”))	Standard Code^b^	4
Alternative and Integrated/ Integrative	(TITLE(“alternative and integrated medicine” OR “alternative integrated medicine” OR “alternative and integrated therapy” OR “alternative integrated therapy” OR “alternative and integrative medicine” OR “alternative integrative medicine” OR “alternative and integrative therapy” OR “alternative integrative therapy” AND NOT “complementary”)) AND PUBYEAR > 1969 AND PUBYEAR < 2014 AND (LIMIT-TO (LANGUAGE, “English”)) AND (LIMIT-TO (SRCTYPE, “j”)) AND (EXCLUDE(DOCTYPE, “er”))	Excludes titles containing “complementary”, in order to prevent other combinations from being searched.	2
Unscientific	(TITLE(“unscientific medicine” OR “unscientific therapy”)) AND PUBYEAR > 1969 AND PUBYEAR < 2014 AND (LIMIT-TO (LANGUAGE, “English”)) AND (LIMIT-TO (SRCTYPE, “j”)) AND (EXCLUDE(DOCTYPE, “er”))	Standard Code^b^	2
Integrated/ Integrative and Alternative	(TITLE(“integrated and alternative medicine” OR “integrated alternative medicine” OR “integrated and alternative therapy” OR “integrated alternative therapy” OR “integrative and alternative medicine” OR “integrative alternative medicine” OR “integrative and alternative therapy” OR “integrative alternative therapy” AND NOT “complementary”)) AND PUBYEAR > 1969 AND PUBYEAR < 2014 AND (LIMIT-TO(LANGUAGE, “English”)) AND (LIMIT-TO(SRCTYPE, “j”)) AND (EXCLUDE(DOCTYPE, “er”))	Excludes titles containing “complementary”, in order to prevent other combinations from being searched.	1
Irregular	(TITLE(“irregular medicine” OR “irregular therapy”)) AND PUBYEAR > 1969 AND PUBYEAR < 2014 AND (LIMIT-TO(LANGUAGE, “English”)) AND (LIMIT-TO(SRCTYPE, “j”)) AND (EXCLUDE(DOCTYPE, “er”))	Standard Code^b^	0
Non-Mainstream	(TITLE(“non-mainstream medicine” OR “non-mainstream therapy”)) AND PUBYEAR > 1969 AND PUBYEAR < 2014 AND (LIMIT-TO (LANGUAGE, “English”)) AND (LIMIT-TO (SRCTYPE, “j”)) AND (EXCLUDE(DOCTYPE, “er”))	Standard Code^b^	0
TOTALS	--	--	7016 (excluding folk and adjunctive)

^a^The resulting articles for “Folk Medicine” were more closely related to indigenous medicines, while the vast majority of resulting articles for “Adjunctive Medicine” focused on adjunctive conventional medicines, hence both these terms were not synonymous with unconventional and were excluded from the remainder of this study

^b^Terms denoted as a standard code are those coded with no exclusions or limitations beyond those associated with the inclusion and exclusion criteria of the study. The standard code is as follows: (TITLE("**[TERM]** medicine" OR “**[TERM]** therapy”)) AND PUBYEAR > 1969 AND PUBYEAR < 2014 AND (LIMIT-TO (LANGUAGE, "English")) AND (LIMIT-TO(SRCTYPE, "j")) AND (EXCLUDE(DOCTYPE, "er"))

The Scopus database is very comprehensive in its coverage of scientific, technical, medical, and social scientific literature [25], which makes it particularly ideal in recovering unconventional medicine-related peer-reviewed articles. Scopus is the largest database of peer-reviewed literature, currently housing 53 million records, including coverage of the entire MEDLINE and EMBASE databases [24]. Scopus is also more comprehensive with regards to its cited reference countsthan Web of Science [25].

Preliminary Scopus searches identified five terms as the most commonly-used: “alternative”, “unconventional”, “complementary”, “complementary and alternative” and “integrated/integrative”. These terms were chosen because a sufficient quantity of articles were cited highly enough to suggest that the papers associated with each term were influential and could be used to establish general trends associated with the changes and meanings of the respective terms. The final Scopus search using these five terms, excluding duplicates, yielded 7012 articles.

**Fig. 1:**
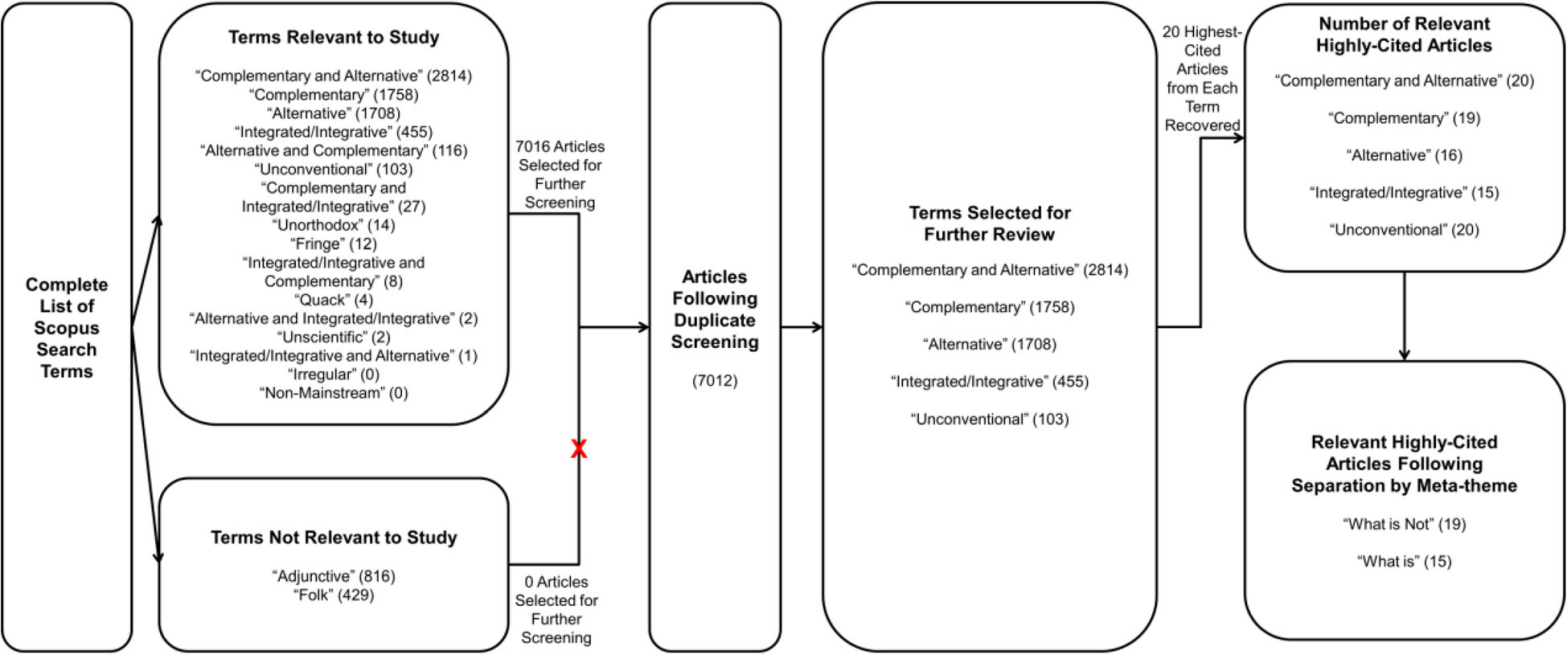
Overview of the Search, Screening and Analysis of Articles in Study

Articles were selected for further review in this study if they met all of the following inclusion criteria: (1) they were obtained from the peer-reviewed literature and; (2) they were applicable to the Canadian or American health care setting; and (3) they were published in English. For criteria (2), this includes Canadian or American publications, and also any other English-language publications as they may have influenced the North American health care system (e.g., are highly-cited in the Canadian or American literature). We limited our sample for close analysis to the 20 most highly-cited articles (and sources they used for their definitions of terms) per search term, because a high number of citations suggests that these articles have had the most influence and impact in shaping the discourse. See Fig. 1. These 100 articles are shown in Table 2.

**Table 2:** The 20 Most Highly-Cited Peer-Reviewed Articles for Five Commonly-Used Unconventional Medicine-Related Search Terms

Term	20 Most Highly-Cited Articles Per Term	
	Full Journal Article Citation	Number of Citations
Complementary and Alternative *N* = 2814 Articles excluded from sample: None	1. Barnes, P. M., Powell-Griner, E., McFann, K., & Nahin, R. L. (2004). Complementary and alternative medicine use among adults: United States, 2002. In *Seminars in Integrative Medicine, 2*(2), 54–71. Philadelphia, PA: W.B. Saunders.	834
	2. Ernst, E. & Cassileth, B. R. (1998). The prevalence of complementary/alternative medicine in cancer. *Cancer*, *83*(4), 777–782.	601
	3. Richardson, M. A., Sanders, T., Palmer, J. L., Greisinger, A., & Singletary, S. E. (2000). Complementary/alternative medicine use in a comprehensive cancer center and the implications for oncology. *Journal of Clinical Oncology*, *18*(13), 2505–2514.	591
	4. Tindle, H. A., Davis, R. B., Phillips, R. S., & Eisenberg, D. M. (2005). Trends in use of complementary and alternative medicine by US adults: 1997–2002. *Alternative Therapies in Health and Medicine*, *11*(1), 42–49.	525
	5. Kronenberg, F., & Fugh-Berman, A. (2002). Complementary and alternative medicine for menopausal symptoms: a review of randomized, controlled trials. *Annals of Internal Medicine*, *137*(10), 805–813.	372
	6. Molassiotis, A., Fernadez-Ortega, P., Pud, D., Ozden, G., Scott, J. A., Panteli, V., … & Patiraki, E. (2005). Use of complementary and alternative medicine in cancer patients: A European survey. *Annals of Oncology*, *16*(4), 655–663.	359
	7. Barnes, P. M., Bloom, B., & Nahin, R. L. (2008). Complementary and alternative medicine use among adults and children: United States, 2007. *National Health Statistics Reports,* (12), 1–23.	327
	8. Boon, H., Stewart, M., Kennard, M. A., Gray, R., Sawka, C., Brown, J. B., … & Haines-Kamka, T. (2000). Use of complementary/alternative medicine by breast cancer survivors in Ontario: Prevalence and perceptions. *Journal of Clinical Oncology*, *18*(13), 2515–2521.	300
	9. Wetzel, M. S., Eisenberg, D. M., & Kaptchuk, T. J. (1998). Courses involving complementary and alternative medicine at US medical schools. *Journal of the American Medical Association*, *280*(9), 784–787.	292
	10. Ernst, E. (2000). Prevalence of use of complementary/alternative medicine: A systematic review. *Bulletin of the World Health Organization*, *78*(2), 258–266.	285
	11. Ni, H., Simile, C., & Hardy, A. M. (2002). Utilization of complementary and alternative medicine by United States adults: Results from the 1999 national health interview survey. *Medical Care*, *40*(4), 353–358.	283
	12. Kessler, R. C., Soukup, J., Davis, R. B., Foster, D. F., Wilkey, S. A., Van Rompay, M. I., & Eisenberg, D. M. (2001). The use of complementary and alternative therapies to treat anxiety and depression in the United States. *American Journal of Psychiatry*, *158*(2), 289–294.	264
	13. Astin, J. A., Marie, A., Pelletier, K. R., Hansen, E., & Haskell, W. L. (1998). A review of the incorporation of complementary and alternative medicine by mainstream physicians. *Archives of Internal Medicine*, *158*(21), 2303–2310.	246
	14. MacLennan, A. H. S. P., Myers, S., & Taylor, A. (2006). The continuing use of complementary and alternative medicine in South Australia: costs and beliefs in 2004. *Medical Journal of Australia*, *184*(1), 27–31.	220
	15. Xue, C. C., Zhang, A. L., Lin, V., Da Costa, C., & Story, D. F. (2007). Complementary and alternative medicine use in Australia: a national population-based survey. *The Journal of Alternative and Complementary Medicine*, *13*(6), 643–650.	208
	16. Harris, P., & Rees, R. (2000). The prevalence of complementary and alternative medicine use among the general population: a systematic review of the literature. *Complementary Therapies in Medicine*, *8*(2), 88–96.	202
	17. Astin, J. A., Pelletier, K. R., Marie, A., & Haskell, W. L. (2000). Complementary and alternative medicine use among elderly persons: One-year analysis of a Blue Shield Medicare supplement. *Journal of Gerontology: Medical Sciences*, *55*(1), M4-M9.	188
	18. Furnham, A., & Forey, J. (1994). The attitudes, behaviors and beliefs of patients of conventional vs. complementary (alternative) medicine. *Journal of Clinical Psychology*, *50*(3), 458–469.	172
	19. Fairfield, K. M., Eisenberg, D. M., Davis, R. B., Libman, H., & Phillips, R. S. (1998). Patterns of use, expenditures, and perceived efficacy of complementary and alternative therapies in HIV-infected patients. *Archives of Internal Medicine*, *158*(20), 2257–2264.	162
	20. Söllner, W., Maislinger, S., DeVries, A., Steixner, E., Rumpold, G., & Lukas, P. (2000). Use of complementary and alternative medicine by cancer patients is not associated with perceived distress or poor compliance with standard treatment but with active coping behavior. *Cancer*, *89*(4), 873–880.	159
Complementary *N* = 1758 Articles excluded from sample: #16	1. Fisher, P., & Ward, A. (1994). Medicine in Europe: Complementary medicine in Europe. *British Medical Journal*, *309*(6947), 107–111.	495
	2. Thomas, K. J., Nicholl, J. P., & Coleman, P. (2001). Use and expenditure on complementary medicine in England: A population based survey. *Complementary Therapies in Medicine*, *9*(1), 2–11.	449
	3. Eisenberg, D. M., Kessler, R. C., Van Rompay, M. I., Kaptchuk, T. J., Wilkey, S. A., Appel, S., & Davis, R. B. (2001). Perceptions about complementary therapies relative to conventional therapies among adults who use both: Results from a national survey. *Annals of Internal Medicine*, *135*(5), 344–351.	399
	4. Ernst, E., & White, A. (2000). The BBC survey of complementary medicine use in the UK. *Complementary Therapies in Medicine*, *8*(1), 32–36.	303
	5. Vincent, C., & Furnham, A. (1996). Why do patients turn to complementary medicine? An empirical study. *British Journal of Clinical Psychology*, *35*(1), 37–48.	284
	6. Downer, S. M., Cody, M. M., McCluskey, P., Wilson, P. D., Arnott, S. J., Lister, T. A., & Slevin, M. L. (1994). Pursuit and practice of complementary therapies by cancer patients receiving conventional treatment. *British Medical Journal*, *309*(6947), 86–89.	249
	7. Rao, J. K., Mihaliak, K., Kroenke, K., Bradley, J., Tierney, W. M., & Weinberger, M. (1999). Use of complementary therapies for arthritis among patients of rheumatologists. *Annals of Internal Medicine*, *131*(6), 409–416.	228
	8. Zollman, C., & Vickers, A. (1999). ABC of complementary medicine: What is complementary medicine? *British Medical Journal*, *319*(7211), 693.	156
	9. Paltiel, O., Avitzour, M., Peretz, T., Cherny, N., Kaduri, L., Pfeffer, R. M., … & Soskolne, V. (2001). Determinants of the use of complementary therapies by patients with cancer. *Journal of Clinical Oncology*, *19*(9), 2439–2448.	146
	10. Zollman, C., & Vickers, A. (1999). ABC of complementary medicine: Users and practitioners of complementary medicine. *British Medical Journal*, *319*(7213), 836.	145
	11. Morris, K. T., Johnson, N., Homer, L., & Walts, D. (2000). A comparison of complementary therapy use between breast cancer patients and patients with other primary tumor sites. *The American Journal of Surgery*, *179*(5), 407–411.	141
	12. Goldbeck-Wood, S., Dorozynski, A., Lie, L. G., Yamauchi, M., Zinn, C., Josefson, D., & Ingram, M. (1996). Complementary medicine is booming worldwide. *British Medical Journal*, *313*(7050), 131–133.	134
	13. Sirois, F. M., & Gick, M. L. (2002). An investigation of the health beliefs and motivations of complementary medicine clients. *Social Science & Medicine*, *55*(6), 1025–1037.	132
	14. Sparber, A., Bauer, L., Curt, G., Eisenberg, D., Levin, T., Parks, S., … & Wootton, J. (2000). Use of complementary medicine by adult patients participating in cancer clinical trials. *Oncology Nursing Forum, 27*(4), 623–630.	132
	15. Pirotta, M. V., Cohen, M. M., Kotsirilos, V., & Farish, S. J. (2000). Complementary therapies: Have they become accepted in general practice? *The Medical Journal of Australia*, *172*(3), 105–109.	125
	16. Vas, J., Méndez, C., Perea-Milla, E., Vega, E., Dolores Panadero, M., León, J. M., … & Jurado, R. (2004). Acupuncture as a complementary therapy to the pharmacological treatment of osteoarthritis of the knee: Randomised controlled trial. *British Medical Journal*, *329*(7476), 1216–1220.	117
	17. Ernst, E., Resch, K. L., & White, A. R. (1995). Complementary medicine: What physicians think of it: A meta-analysis. *Archives of Internal Medicine*, *155*(22), 2405–2408.	116
	18. Ernst, E., Rand, J. I., & Stevinson, C. (1998). Complementary therapies for depression: An overview. *Archives of General Psychiatry*, *55*(11), 1026–1032.	104
	19. Fulder, S., & Munro, R. (1985). Complementary medicine in the United Kingdom: Patients, practitioners, and consultations. *The Lancet*, *326*(8454), 542–545.	104
	20. Nam, R. K., Fleshner, N., Rakovitch, E., Klotz, L., Trachtenberg, J., Choo, R., … & Danjoux, C. (1999). Prevalence and patterns of the use of complementary therapies among prostate cancer patients: an epidemiological analysis. *The Journal of Urology*, *161*(5), 1521–1524.	103
Alternative *N* = 1708 Articles excluded from sample: #4, 15, 17, 19	1. Eisenberg, D. M., Davis, R. B., Ettner, S. L., Appel, S., Wilkey, S., Van Rompay, M., & Kessler, R. C. (1998). Trends in alternative medicine use in the United States, 1990–1997: Results of a follow-up national survey. *Journal of the American Medical Association*, *280*(18), 1569–1575.	4589
	2. Astin, J. A. (1998). Why patients use alternative medicine: Results of a national study. *Journal of the American Medical Association*, *279*(19), 1548–1553.	1729
	3. MacLennan, A. H., Wilson, D. H., & Taylor, A. W. (1996). Prevalence and cost of alternative medicine in Australia. *The Lancet*, *347*(9001), 569–573.	667
	4. McNeil, B. J., Pauker, S. G., Sox Jr, H. C., & Tversky, A. (1982). On the elicitation of preferences for alternative therapies. *New England Journal of Medicine*, *306*(21), 1259–1262.	513
	5. Angell, M., & Kassirer, J. P. (1998). Alternative medicine-the risks of untested and unregulated remedies. *New England Journal of Medicine*, *339*(12), 839–841.	475
	6. Burstein, H. J., Gelber, S., Guadagnoli, E., & Weeks, J. C. (1999). Use of alternative medicine by women with early-stage breast cancer. *New England Journal of Medicine*, *340*(22), 1733–1739.	441
	7. MacLennan, A. H., Wilson, D. H., & Taylor, A. W. (2002). The escalating cost and prevalence of alternative medicine. *Preventive Medicine*, *35*(2), 166–173.	308
	8. Spigelblatt, L., Laîné-Ammara, G., Pless, I. B., & Guyver, A. (1994). The use of alternative medicine by children. *Pediatrics*, *94*(6), 811–814.	282
	9. Lee, M. M., Lin, S. S., Wrensch, M. R., Adler, S. R., & Eisenberg, D. (2000). Alternative therapies used by women with breast cancer in four ethnic populations. *Journal of the National Cancer Institute*, *92*(1), 42–47.	256
	10. Kaptchuk, T. J. (2002). The placebo effect in alternative medicine: Can the performance of a healing ritual have clinical significance? *Annals of Internal Medicine*, *136*(11), 817–825.	254
	11. Heck, A. M., Dewitt, B. A., & Lukes, A. L. (2000). Potential interactions between alternative therapies and warfarin. *American Journal of Health-System Pharmacy*, *57*(13), 1221–1227.	232
	12. Paramore, L. C. (1997). Use of alternative therapies: Estimates from the 1994 Robert Wood Johnson Foundation national access to care survey. *Journal of Pain and Symptom Management*, *13*(2), 83–89.	205
	13. Kelner, M., & Wellman, B. (1997). Health care and consumer choice: Medical and alternative therapies. *Social Science & Medicine*, *45*(2), 203–212.	188
	14. Fontanarosa, P. B., & Lundberg, G. D. (1998). Alternative medicine meets science. *Journal of the American Medical Association*, *280*(18), 1618–1619.	187
	15. Das, D. K., & Maulik, N. (2006). Resveratrol in cardioprotection: A therapeutic promise of alternative medicine. *Molecular Interventions*, *6*(1), 36.	157
	16. Newton, K. M., Buist, D. S., Keenan, N. L., Anderson, L. A., & LaCroix, A. Z. (2002). Use of alternative therapies for menopause symptoms: Results of a population-based survey. *Obstetrics & Gynecology*, *100*(1), 18–25.	150
	17. Trenk, D., Stone, G. W., Gawaz, M., Kastrati, A., Angiolillo, D. J., Müller, U., … & Neumann, F. J. (2012). A randomized trial of prasugrel versus clopidogrel in patients with high platelet reactivity on clopidogrel after elective percutaneous coronary intervention with implantation of drug-eluting stents: Results of the TRIGGER-PCI (Testing Platelet Reactivity In Patients Undergoing Elective Stent Placement on Clopidogrel to Guide Alternative Therapy With Prasugrel) study. *Journal of the American College of Cardiology*, *59*(24), 2159–2164.	149
	18. Ernst, E., & Pittler, M. H. (1997). Alternative therapy bias. *Nature*, *385*(6616), 480.	147
	19. Meeker, W. C., & Haldeman, S. (2002). Chiropractic: A profession at the crossroads of mainstream and alternative medicine. *Annals of Internal Medicine*, *136*(3), 216–227.	145
	20. Unützer, J., Klap, R., Sturm, R., Young, A. S., Marmon, T., Shatkin, J., & Wells, K. B. (2000). Mental disorders and the use of alternative medicine: Results from a national survey. *American Journal of Psychiatry*, *157*(11).	144
Integrated/ Integrative *N* = 455 Articles excluded from sample: #7, 9, 12, 14, 15	1. Bell, I. R., Caspi, O., Schwartz, G. E., Grant, K. L., Gaudet, T. W., Rychener, D., … & Weil, A. (2002). Integrative medicine and systemic outcomes research: Issues in the emergence of a new model for primary health care. *Archives of Internal Medicine*, *162*(2), 133–140.	146
	2. Kligler, B., Maizes, V., Schachter, S., Park, C. M., Gaudet, T., Benn, R., … & Remen, R. N. (2004). Core competencies in integrative medicine for medical school curricula: A proposal. *Academic Medicine*, *79*(6), 521–531.	91
	3. Snyderman, R., & Weil, A. T. (2002). Integrative medicine: Bringing medicine back to its roots. *Archives of Internal Medicine*, *162*(4), 395–397.	76
	4. Girman, A., Lee, R., & Kligler, B. (2003). An integrative medicine approach to premenstrual syndrome. *American Journal of Obstetrics and Gynecology*, *188*(5), S56-S65.	51
	5. Wang, J., & Xiong, X. (2012). Current situation and perspectives of clinical study in integrative medicine in China. *Evidence-Based Complementary and Alternative Medicine*.	47
	6. Xu, H., & Chen, K. (2008). Integrative medicine: The experience from China. *The Journal of Alternative and Complementary Medicine*, *14*(1), 3–7.	41
	7. Scullin, C., Scott, M. G., Hogg, A., & McElnay, J. C. (2007). An innovative approach to integrated medicines management. *Journal of Evaluation in Clinical Practice*, *13*(5), 781–788.	41
	8. Edelman, D., Oddone, E. Z., Liebowitz, R. S., Yancy, W. S., Olsen, M. K., Jeffreys, A. S., … & Gaudet, T. W. (2006). A multidimensional integrative medicine intervention to improve cardiovascular risk. *Journal of General Internal Medicine*, *21*(7), 728–734.	41
	9. Hellström, L. M., Bondesson, Å., Höglund, P., Midlöv, P., Holmdahl, L., Rickhag, E., & Eriksson, T. (2011). Impact of the Lund Integrated Medicines Management (LIMM) model on medication appropriateness and drug-related hospital revisits. *European Journal of Clinical Pharmacology*, *67*(7), 741–752.	39
	10. Maizes, V., Rakel, D., & Niemiec, C. (2009). Integrative medicine and patient-centered care. *Explore: The Journal of Science and Healing*, *5*(5), 277–289.	39
	11. Sundberg, T., Halpin, J., Warenmark, A., & Falkenberg, T. (2007). Towards a model for integrative medicine in Swedish primary care. *BMC Health Services Research*, *7*(1), 107.	38
	12. Kidd, P. M. (2002). Autism, an extreme challenge to integrative medicine. Part 1: The knowledge base. *Alternative Medicine Review*, *7*(4), 292–316.	38
	13. Gaudet, T. W. (1998). Integrative medicine: The evolution of a new approach to medicine and to medical education. *Integrative Medicine*, *1*(2), 67–73.	38
	14. Kidd, P. M. (2002). Autism, an extreme challenge to integrative medicine. Part II: Medical management. *Alternative Medicine Review*, *7*(6), 472–499.	37
	15. Bergkvist, A., Midlöv, P., Höglund, P., Larsson, L., Bondesson, Å., & Eriksson, T. (2009). Improved quality in the hospital discharge summary reduces medication errors—LIMM: Landskrona Integrated Medicines Management. *European Journal of Clinical Pharmacology*, *65*(10), 1037–1046.	35
	16. Hsiao, A. F., Ryan, G. W., Hays, R. D., Coulter, I. D., Andersen, R. M., & Wenger, N. S. (2006). Variations in provider conceptions of integrative medicine. *Social Science & Medicine*, *62*(12), 2973–2987.	34
	17. Weil, A. (2000). The significance of integrative medicine for the future of medical education. *American Journal of Medicine*, *108*(5), 441–443.	31
	18. Lu, A. P., & Chen, K. J. (2009). Integrative medicine in clinical practice: From pattern differentiation in traditional Chinese medicine to disease treatment. *Chinese Journal of Integrative Medicine*, *15*(2), 152–152.	29
	19. Bell, I. R., Cunningham, V., Caspi, O., Meek, P., & Ferro, L. (2004). Development and validation of a new global well-being outcomes rating scale for integrative medicine research. *BMC Complementary and Alternative Medicine*, *4*(1), 1–10.	29
	20. Wang, J., Yao, K., Yang, X., Liu, W., Feng, B., Ma, J., … & Xiong, X. (2012). Chinese patent medicine liu wei di huang wan combined with antihypertensive drugs, a new integrative medicine therapy, for the treatment of essential hypertension: A systematic review of randomized controlled trials. *Evidence-Based Complementary and Alternative Medicine*.	28
Unconventional *N* = 103 Articles excluded from sample: None	1. Eisenberg, D. M., Kessler, R. C., Foster, C., Norlock, F. E., Calkins, D. R., & Delbanco, T. L. (1993). Unconventional medicine in the United States--prevalence, costs, and patterns of use. *New England Journal of Medicine*, *328*(4), 246–252.	2788
	2. Druss, B. G., & Rosenheck, R. A. (1999). Association between use of unconventional therapies and conventional medical services. *Journal of the American Medical Association*, *282*(7), 651–656.	310
	3. Kelly, K. M., Jacobson, J. S., Kennedy, D. D., Braudt, S. M., Mallick, M., & Weiner, M. A. (2000). Use of unconventional therapies by children with cancer at an urban medical center. *Journal of Pediatric Hematology/Oncology*, *22*(5), 412–416.	109
	4. Menniti-Ippolito, F., Gargiulo, L., Bologna, E., Forcella, E., & Raschetti, R. (2002). Use of unconventional medicine in Italy: A nation-wide survey. *European Journal of Clinical Pharmacology*, *58*(1), 61–64.	104
	5. Ernst, E. (2003). Serious adverse effects of unconventional therapies for children and adolescents: A systematic review of recent evidence. *European Journal of Pediatrics*, *162*(2), 72–80.	100
	6. Eidinger, R. N., & Schapira, D. V. (1984). Cancer patients' insight into their treatment, prognosis, and unconventional therapies. *Cancer*, *53*(12), 2736–2740.	88
	7. Vickers, A., Cassileth, B., Ernst, E., Fisher, P., Goldman, P., Jonas, W., … & Silagy, C. (1997). How should we research unconventional therapies? A panel report from the Conference on Complementary and Alternative Medicine Research Methodology, National Institutes of Health. *International Journal of Technology Assessment in Health Care*, *13*(1), 111–121.	77
	8. Vickers, A. J., & Cassileth, B. R. (2001). Unconventional therapies for cancer and cancer-related symptoms. *The Lancet Oncology*, *2*(4), 226–232.	75
	9. Nayak, S., Matheis, R. J., Schoenberger, N. E., & Shiflett, S. C. (2003). Use of unconventional therapies by individuals with multiple sclerosis. *Clinical Rehabilitation*, *17*(2), 181–191.	74
	10. Moser, G., Tillinger, W., Sachs, G., Maier-Dobersberger, T., Wyatt, J., Vogelsang, H., … & Gangl, A. (1996). Relationship between the use of unconventional therapies and disease-related concerns: A study of patients with inflammatory bowel disease. *Journal of Psychosomatic Research*, *40*(5), 503–509.	67
	11. Campion, E. W. (1993). Why unconventional medicine? *New England Journal of Medicine*, *328*(4), 282.	53
	12. Dalen, J. E. (1998). Conventional and unconventional medicine: Can they be integrated? *Archives of Internal Medicine*, *158*(20), 2179–2181.	48
	13. Bold, J., & Leis, A. (2001). Unconventional therapy use among children with cancer in Saskatchewan. *Journal of Pediatric Oncology Nursing*, *18*(1), 16–25.	46
	14. Resch, K. I., Ernst, E., & Garrow, J. (2000). A randomized controlled study of reviewer bias against an unconventional therapy. *Journal of the Royal Society of Medicine*, *93*(4), 164–167.	43
	15. Blumberg, D. L., Grant, W. D., Hendricks, S. R., Kamps, C. A., & Dewan, M. J. (1995). The physician and unconventional medicine. *Alternative Therapies in Health and Medicine*, *1*(3), 31–35.	43
	16. Gaus, W., & Hoegel, J. (1995). Studies on the efficacy of unconventional therapies. Problems and designs. *Arzneimittel-Forschung*, *45*(1), 88–92.	40
	17. Lewith, G. T., & Watkins, A. D. (1996). Unconventional therapies in asthma: An overview. *Allergy*, *51*(11), 761–769.	38
	18. Kappauf, H., Leykauf-Ammon, D., Bruntsch, U., Horneber, M., Kaiser, G., Büschel, G., & Gallmeier, W. M. (2000). Use of and attitudes held towards unconventional medicine by patients in a department of internal medicine/oncology and haematology. *Supportive Care in Cancer*, *8*(4), 314–322.	37
	19. Kaegi, E. (1998). Unconventional therapies for cancer: 1. Essiac. *Canadian Medical Association Journal*, *158*(7), 897–902.	37
	20. Kaegi, E. (1998). Unconventional therapies for cancer: 2. Green tea. *Canadian Medical Association Journal*, *158*(8), 1033.	33

**Note:** Citation count for each article was recorded on September 11, 2014

### Data analysis

Each article was coded with the goal of obtaining phrases, sentences, or paragraphs of text that supported the developing categories and themes and investigated the debates present in the sample [22].

Gieryn’s theory was used to guide the coding process, including both the development of the content categories, and the subsequent acquisition of phrases, sentences or paragraphs of text that supported these themes developed from these categories. Gieryn’s [13] perspective highlighted the need to investigate whether and how authors may use the rhetoric of science to their advantage (or disadvantage) in labelling and describing the concept of unconventional medicine. For example, in introducing or changing a term, did the author(s) appear to refer to science as an intellectual authority in order to distinguish a group or practice as (more) scientific than a competitor?

All text excerpts were first categorized into topics organized into series of themes, and following this, different excerpts within the same theme were re-read again to screen for duplication of ideas. Duplicated ideas were then reduced, resulting in all remaining texts to be indicated of a larger volume of texts, used as exemplars in interpreting the findings of the textual analysis [22].

## Results

The five most commonly-used terms in articles published between 1975 and 2013 were as follows, from most to least used in article titles: “complementary and alternative”, “complementary”, “alternative”, “integrated/integrative”, and “unconventional”. The order of the first instances of these terms being used with respect to the meaning of unconventional medicine, is as follows: “alternative” (1975) [26], “unconventional” (1980) [27], “complementary” (1984) [28], “complementary and alternative” (1994) [29], and “integrated/integrative” (1995) [30].

**Fig. 2:**
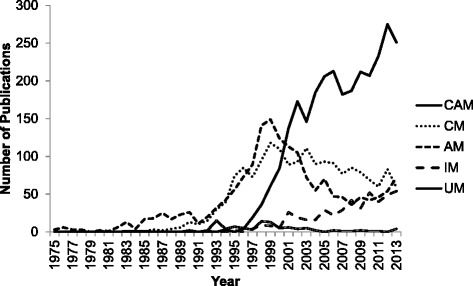
Number of Publications with Unconventional Medicine-Related Terms in Title per Year from 1975–2013

No articles recovered from Scopus that had titles including one of the five commonly used unconventional medicine-related terms were published prior to 1975. “alternative” was the solely used term between 1975 and 1983, and remained the predominantly used term until 1990 and again between 1997 and 1999. The use of the term “complementary” increased in the early to mid-1990s, and its use has remained relatively consistent up until 2013. The use of the combination term “complementary and alternative” increased sharply beginning in 2000, and continues to be the most commonly used term up to 2013. The use of the term “integrated/integrative” began in the mid 1990s, and its use has since slowly increased each year. “Unconventional” is a term used generally less frequently than the other four terms, though its use has been continuous between 1980 and 2013. Its use experienced a small rise between the mid-1990s until the early 2000s. The number of publications associated with these unconventional medicine-related terms tracked per year is shown in Fig. 2. There were no significant changes in the meaning of individual terms over time in the literature analysed.

During the textual analysis, we found that the meanings associated with the terms “alternative”, “unconventional”, “complementary” and “complementary and alternative” differed greatly from the term “integrated/integrative”. While the first four terms were often used by authors interchangeably and possessed similar definitions, the use of the term “integrated/integrative” stood out as unique. These first four terms were characterized by being defined by “what is not”, including being listed or grouped as items *not* part of conventional medicine, directly being categorized as therapies or interventions *not* taught/used in conventional medicine, used to treat therapy demands that are *not* met by conventional medicine, and therapies *lacking in* research on safety, efficacy and effectiveness. In contrast, the fifth term was characterized oppositely as by “what is”, including a new model or system of healthcare, the combination of conventional and unconventional medicine, accounting for the whole person, and preventative maintenance of health.

Importantly, it should be noted that all themes identified in this study were evaluated by the year in which the respective article was published. No significant changes in the appearance or disappearance of any of the “what is not” or the “what is” themes or subthemes occurred over this study’s timeframe. However, it should be noted that all peer-reviewed articles from which subthemes were extracted were published in 1993 or later. The two major themes each containing four subthemes are the focus of this section.

### “Alternative”, “unconventional”, “complementary” and “complementary and alternative” medicine: an analysis of terms defined by “what they are not”

Defining terms by “what is not” comprises one of two major themes within this study. The definitions of four terms – “alternative”, “unconventional”, “complementary” and “complementary and alternative” – can generally be described as “what is not” definitions. Authors using any or all of these terms in their articles consistently defined them as something that *was not* within the realm of conventional medicine. While defining by “what is not” served as an overarching theme, within it there were four subthemes that emerged upon evaluating the texts relevant to this group of authors.

The first sub-theme that emerged was the fact that many authors either defined or used these terms in reference to a list or group of therapies or interventions:

“The most common CAM [complementary and alternative medicine] interventions/therapies included in the surveys, in order of most common inclusion, were chiropractic care, acupuncture, herbal medicine, hypnosis, massage therapy, relaxation techniques, biofeedback, and homeopathic treatment. CAM interventions/therapies such as chelation therapy, energy therapies, qi gong, tai chi, yoga, high-dose vitamins, and spirituality/prayer for health purposes were less commonly included.” ([31], p. 64)

While not typically definitions themselves, such lists and/or groups of items often defined the parameters of these therapies in studies. These lists or groups of therapies were often accompanied with a given definition, explaining the reasoning behind what was selected for inclusion in the list.

The second theme related to a major reason as to why these therapies were found on these lists of groups. The best example of this is the definition of the term “unconventional therapies” provided in Eisenberg et al.’s ([5], p. 246) study which is also the most highly-cited definition among the 80 papers [1, 32–43]:

Medical interventions **neither taught widely at U.S. medical schools or generally available in US hospitals**.” (emphasis added)

The third subtheme involved describing these as therapies that were being used to meet demands *not* met by conventional medicine. One good example of this is yet another highly-cited definition [40, 44–47] provided by Ernst et al. ([48], p. 506):

“**Diagnosis, treatment and/or prevention which complements mainstream medicine** by contributing to a common whole, **by satisfying a demand not met by orthodoxy** or by diversifying the conceptual frameworks of medicine” (emphasis added)

The fourth subtheme captured the notion that there is a lack of academic research surrounding the safety, efficacy and/or effectiveness of unconventional therapies. Authors defined “complementary”, “alternative”, complementary and alternative” or unconventional” medicine as therapies *not* having sufficient evidence for use:

“Providers, methods and modes of diagnostics, treatment and/or prevention, for which, **without sound evidence, specificity, sensitivity and/or therapeutic efficacy** is commonly claimed in respect to a definite medical problem.” (emphasis added) ([49], p. 315)

Many authors who used these four terms made clear that they felt there was a need to rigorously evaluate unconventional therapies. Many included a call for increased research and funding to be devoted to the study of this subject area, to ensure that unconventional medicines met a certain standard of safety and efficacy:

“In light of these observations, we suggest that federal agencies, private corporations, foundations, and academic institutions adopt a more proactive posture concerning the implementation of clinical and basic science research, the development of relevant educational curricula, credentialing and referral guidelines, improved quality control of dietary supplements, and the establishment of postmarket surveillance of drug-herb (and drug supplement) interactions.” ([37], p. 1575)

“Alternative treatments should be subjected to scientific testing no less rigorous than that required for conventional treatments.” ([32], p. 841

### “Integrated/integrative” medicine: an analysis of terms defined by “what is”

In contrast, authors who used the term “integrated/integrative”, only defined it in a way that it could exist independently, as opposed to by defining it by something that is “not”, and therefore, we argue that these authors defined this term by “what is”.

There were also four subthemes that could be identified from among this group of authors solely using the term “integrated/integrative”.

The first subtheme involves promoting a new model or system of healthcare. The authors who used this term advocated for fundamental changes to the conventional healthcare system. For example:

“Integrative medicine is not a radical movement, but it can produce major change. Its point is to position medicine in such a way that it can continue to build on its fundamental platform of science and at the same time reposition itself to create a health care system that more broadly focuses on the well-being of patients as well as practitioners.” ([9], p. 397)

The second theme related to the thoughtful combination of conventional and unconventional therapies. Largely, the authors who wrote about “integrated/integrative” medicine believed that numerous and positively-perceived aspects of unconventional medicines could complement conventional ones to form a novel, ameliorated healthcare system. Their language often included a criticism of the reductionist form of conventional medicine-based research, and the need to adopt research strategies that would showcase the optimal therapeutic ability of unconventional therapies. For example:

“The challenge is to sort through all the evidence about all healing systems and try to **extract those ideas and practices that are useful, safe, and cost-effective. Then we must try to merge them into a new, comprehensive system of practice** that has an evidence base and also address consumer demands. The most appropriate term for this new system is integrative medicine.” (emphasis added) ([50], p. 442)

“We believe that the health care system must be reconfigured to restore the primacy of caring and the patient-physician relationship, to promote health and healing as well as treatment of disease, and **to take account of the insufficiency of science and technology alone to shape the ideal practice of medicine**. The new design must also incorporate compassion, promote the active engagement of patients in their care, and **be open to what are now termed complementary and alternative approaches to improve health and well-being.**” (emphasis added) ([9], p. 396)

“A published case study of communication has shown that IM [integrative medicine] panel members representing a wide range of theories of health and healing were able to communicate easily with one another, **when they limited themselves to the scientific language of biomedicine**.” (emphasis added) [51]

The third theme in this category is that of accounting for the whole person. This idea was discussed frequently in these articles, and involved taking into account the different “dimensions” of a patient, including their biological, psychological, sociological and spiritual factors, as a practitioner in this new integrative medical model. Furthermore, many of these authors claimed that by accounting for each of these factors, a clinician’s quality of practice would also improve:

“Integrative medicine represents a higher-order system of systems of care that **emphasizes wellness and healing of the entire person (bio-psycho-socio-spiritual dimensions)** as primary goals, drawing on both conventional and CAM approaches in the context of a supportive and effective physician-patient relationship.” (emphasis added) ([52], p. 133)

“**All factors that influence health, wellness and disease are taken into consideration, including mind, spirit and community, as well as body.** These multiple influences on health have been firmly documented in the literature but are not often recognized as important in medical practice. Conventional medical care tends to focus on the physical influences on health. **An integrative approach also addresses the importance of the nonphysical (eg, emotions, spirit, social) influences on physical health and disease.**” (emphasis added) ([53], p. 279)

Lastly, the fourth theme associated with the term “integrative/integrated” is regarding the preventative maintenance of health. The final theme commonly discussed by this group of authors involved the idea that, instead of waiting to treat disease once it developed, the focus of healthcare should be shifted towards addressing preventative health in both patients and healthcare providers:

“In addition to providing the best conventional care, integrative medicine focuses on **preventive maintenance of health** by paying attention to all relative components of lifestyle, including diet, exercise, stress management, and emotional well-being.” (emphasis added) ([9], p. 396).

Authors highlighted that preventative maintenance of health also included the teaching of physicians to care for themselves. These authors advocated for medical schools to encourage their students to follow healthy lifestyles.

## Discussion

The purpose of conducting this analysis was to explore how the meanings associated with unconventional medicine-related terms have changed over time in the peer-reviewed literature. We had expected to find an evolution in use of these terms and their meanings over time. Instead, a key finding was that four out of five terms selected for further analysis – “alternative”, “unconventional”, “complementary” and “complementary and alternative” – were frequently defined and/or used interchangeably. While the use and definition of the fifth term, “integrated/integrative”, was so unique that it would be incorrect to categorize it as synonymous with the other terms as we had originally assumed when embarking on the study. In addition to this dichotomy between “what is not” versus “what is” in unconventional medicine-related terminology, another key area of relevance relates to Thomas Gieryn’s [13] scientific boundary work described earlier in this paper. These two key findings make up the two major points of discussion in the next section.

### The divide between “what is” vs. “what is not”

The first key finding relates to the existence of two distinct groups of authors in the literature including those who use terms defined by “what is not” (i.e. “alternative”, “unconventional”, “complementary” and “complementary and alternative”), and those who use the term defined by “what is” (i.e. “integrated/integrative”). More importantly, a key difference in stance is what separates these two groups of authors.

Those defining terms by “what is not” generally did not seek to make changes in the sociopolitical positioning of unconventional medicine. In addition to not focussing attention on which of the four terms they used, these authors aimed to position themselves as a group who viewed and evaluated unconventional therapies “objectively”. These authors frequently explained that their reason for conducting research into unconventional therapies was to fill in knowledge gaps in the conventional medicine community. “What is not” authors often advocated for greater scientific research of unconventional therapies with a stated goal of determining the safety and efficacy, among other aspects, of such therapies.

In contrast, those defining terms by “what is” sought to evaluate certain unconventional therapies that, if deemed to have sufficient evidence for their use, should then be offered within an integrative medical model. The “what is” group of authors were explicit in stating that their goal lies in reforming the current conventional healthcare system in two major ways: revising medical education and changing the way medicine is practiced. Authors in this group also advocated for teaching medical students and practitioners how to lead more healthy lifestyles during their training and practice respectively. “What is” authors also engaged in an interesting form of advocacy, as they made claims that their proposed changes in conventional healthcare would serve as a solution to global health crises. By addressing an issue that has been highlighted in major reports including those published by the Lancet Commission and the World Health Organization, among other influential sources, these authors argued that their suggested reforms of the healthcare system are needed to keep pace with population demands in health care [54, 55].

Stakeholders (such as funding bodies, academic journals and unconventional medicine-related care centres or educational institutions) interested in attracting authors with an interest in this field appear to have adjusted their language to appeal to both groups. For example, the National Center for Complementary and Alternative Medicine (NCCAM) (formerly named as the Office of Alternative Medicine (OAM)), changed its name to the National Center for Complementary and Integrative Health (NCCIH) in December 2014 [56]. Prior to making a decision on its new name, the NCCIH invited public comment allowing anyone with an interest to contribute to the idea of their proposed name change. This appears to deliberately signal openness to funding studies from individuals in both the “what is not” and “what is” groups. It should be noted that this strategy of including terminology used by both groups is not unique to the NCCIH, as the deliberate combination of terms is also increasingly being seen in peer-reviewed journal names (i.e., Journal of Complementary and Integrative Medicine [http://www.degruyter.com/view/j/jcim], Alternative and Integrative Medicine [http://esciencecentral.org/journals/alternative-integrative-medicine.php]), practitioner associations (i.e., Association of Complementary and Integrative Physicians of British Columbia [http://www.acpbc.org/]) and research/practice centres (i.e., Australian Research Centre in Complementary and Integrative Medicine [http://www.uts.edu.au/research-and-teaching/our-research/arccim], Mayo Clinic’s Complementary and Integrative Medicine Program [http://www.mayo.edu/research/centers-programs/complementary-integrative-medicine/complementary-integrative-medicine-program/overview]), that exist in this field.

### The use of scientific rhetoric for legitimacy

The second key finding revolves around scientific rhetoric, which relates to Gieryn’s [13] scientific boundary work. Despite differences that exist between both groups of authors, each focussed on the importance of science in defining medicine that was not “conventional”. Authors defining terms by “what is not” used scientific rhetoric to identify the importance of further research addressing the safety and efficacy of these therapies. For example, Eisenberg et al. called for academic institutions to implement further “clinical and basic science research” regarding unconventional therapies ([37], p. 1575), while Angell and Kassirer stated that unconventional medicine has not been “scientifically tested”, and argue that such therapies should undergo “scientific testing” equally rigorous to that required for conventional medicines [32, p. 839, 841] thus ruling them “out” of the realm of conventional medicine for now.

Authors defining terms by “what is” used this rhetoric to describe their integration strategy, which depended upon scientific evidence. Snyderman and Weil explained that the point of integrative medicine is to align medicine so as to allow for it to continue building on its “fundamental platform of science” ([9], p. 397). Bell et al. argued elsewhere in their article that it is mandatory that integrative medicine be based in “good science”, and that integrative medicine values “scientific evidence” as a way of enhancing the public’s understanding of good health as a society ([52], p. 135). Finally, Sundberg et al. explained with regard to an integrative medicine clinic involved in their study, that integrative medicine members comprised of many different theories of health/healing, could easily convey information with one other, when they restricted communication to the “scientific language of biomedicine” [51]. Thus, rather than focusing on scientific rhetoric to rule therapies “out”, they leverage scientific notions of validity and legitimacy to justify the integration of unconventional therapies into conventional medicine, thereby ruling them “in.” This is a subtle difference in rhetorical strategy, but it signifies a different project altogether, and one of which many users of these terminologies may not be aware.

### Study limitations

The primary limitation that should be addressed in this study is with regards to potentially unaccounted for, yet influential, discourses emerging in the most recent years of this study’s timeframe. The nature of the peer-reviewed publication process involves a lag time between authors reading influential work, incorporating it into their studies, and ultimately citing/publishing this work. To combat this limitation, efforts were made to search both the online and grey literature sources to determine whether any discourses surrounding unconventional medicine-related terms appeared to influence the time period corresponding with the years including and directly preceding 2013. No new themes were identified using this strategy.

### Study implications

From a theoretical standpoint, our study serves as a compelling example of Gieryn’s perspective on scientific boundary work. We have demonstrated that scientific rhetoric is evident in the discourses of both “what is not” and “what is” author groups, which demarcate their work within a conventional medicine framework from that of work performed by members of unconventional medicine. Through applying Gieryn’s theory, we identify two distinct ways in which conventional practitioners have constructed boundaries to demarcate their work and research.

This study provides an analysis of the labelling regarding unconventional medicine-related terms, potentially facilitating more nuanced use of these key terms by the stakeholders invested in the field of unconventional medicine. This study has illuminated a tension in the texts published in the medical literature about unconventional medicine in which two groups of authors leverage scientific rhetoric, but to very different ends. Additionally, our findings can help to explain why academics or practitioners who work in an environment primarily categorized by supporters of “what is not” language but who use “what is” language (or vice-versa) may face criticism from their colleagues, or a lack of traction, with regards to their research or practice goals within the field of unconventional medicine. Lastly, this study suggests that authors looking to continue (or begin) publishing their work in this field, should recognize that these five commonly-used unconventional medicine-related terms are not synonymous in meaning or definition.

### Future directions

This study was designed to analyse discourses within the peer-reviewed medical literature, so it was not surprising that the majority of texts were dominated by the voice of conventional medical practitioners and researchers. This was the case in both the “what is not” and “what is” groups of articles. This study was not designed to adequately capture the voices of unconventional medicine members, which were almost completely non-existent in the peer-reviewed literature.

Follow-up studies should seek to understand discourses generated by unconventional practitioners and patients. This may involve evaluating other forms of media unconventional medicine practitioners use to produce their discourses. It will be important to explore how unconventional medicine practitioners describe themselves and how patients are interpreting the differing discourses of unconventional medicine.

Furthermore, this study focused on the peer-reviewed literature. Future studies should attempt to understand discourses found in forms of media inclusive of the grey literature or the internet to determine whether our results are comparable. By doing this, such information obtained may help to explain whether both “what is not” and “what is” authors engage, if at all, using similar or different discourses outside of the realm of peer-reviewed media. Understanding what and who leads the creation of discourses surrounding the naming of unconventional medicine members found in other forms of media may also help uncover key information associated with the dynamics between and within practitioners and researchers of both conventional and unconventional medical professions.

## Conclusions

This study identified a dichotomy between two groups of conventional medicine practitioners. The first group described the terms “alternative”, “unconventional”, “complementary” and “complementary and alternative” as therapies that are *not* taught/used in conventional medicine, meet needs *not* addressed in conventional medicine, and have *not* been shown to be safe or effective. The second group defined the term “integrated/integrative” medicine as a new model of health and wellness that advocates combination of conventional and evidence-based unconventional therapies, accounts for the whole person and promotes the preventative maintenance of health in both patient and practitioner.

From a practical standpoint, this research has served to crystallize a debate that has been played out in the texts written by both groups of authors. Very different language use between these two author types suggests the possibility of a growing intra-professional split among this group of seemingly homogeneous conventional medicine members. This study will sensitize its audience to a greater awareness of naming surrounding unconventional medicine-related terms and how they may be interpreted by others within their field. This study can help to explain why academics or practitioners who work in an environment primarily consisting of supporters of “what is not” language but who use “what is” language (or vice-versa) may face criticism from their colleagues, or a lack of traction, with regards to their research or practice goals within the field of unconventional medicine. Lastly, this study’s finding of a clear dichotomy in language and meaning associated with “what is not” versus “what is” language provides a service to authors looking to continue (or begin) publishing their work in this field, helping them to identify that these five commonly-used unconventional medicine-related terms are not simply synonymous in meaning or definition.

While both groups incorporate scientific rhetoric into their discourses, each seeks to advance their own distinct agenda. The “what is not” group fundamentally attempts to study the field of unconventional medicine as objective, academic researchers. In contrast, the “what is” group are activists with an interest in reforming the present model of conventional healthcare training and delivery. This divide between these two groups of authors within conventional medicine will require ongoing investigation as the discourses change over time.

## Ethics approval and consent to participate

This study involved the peer-reviewed literature only; it did not require ethics approval or consent to participate.

## Consent for publication

All authors consent to this manuscript’s publication.

## Abbreviations

CAM: Complementary and Alternative Medicine
NCCAM: National Center for Complementary and Alternative Medicine
NCCIH: National Center for Complementary and Integrative Health
OAM: Office of Alternative Medicine
